# Recognising and supporting authentic learning in a changing world: the opportunities and threats of AI

**DOI:** 10.1038/s41415-024-7948-9

**Published:** 2024-10-25

**Authors:** Francesca Mullan, Helen Mather, Heidi Bateman, Alison Cairns, Melanie Nasseripour, Viv Binnie, Luke Dawson, Giles McCracken, Janice Ellis

**Affiliations:** 41415199222001 Newcastle University, School of Dental Sciences, UK; 41415199222002 Faculty of Dentistry, Oral and Craniofacial Sciences, King´s College London, UK; 41415199222003 University of Glasgow, Dental School, UK; 41415199222004 University of Liverpool, Dental School, UK

## Abstract

Since the term AI (artificial intelligence) was first coined, it has become embedded in modern life, with debate focusing on its challenges. In dentistry, AI is being used in clinical and education practice; however, many educators have limited knowledge or skills in its use. The British Alliance of Researchers in Dental Education and Scholarship hosted an AI-themed conference in November 2023. The conference organisers set out to initiate discussion on the use of AI in UK dental education, including a focused workshop to develop a consensus opinion. Before the conference, potential opportunities and threats associated with AI were determined, and through a pre-conference questionnaire, these were prioritised for in-depth discussion. During the workshop, personalised learning, support for learning, educator resources and equality were all identified as opportunities presented by AI, while digital literacy, misuse and safety were seen as potential threats. Two key overarching concepts emerged at the end of the conference: recognition that AI is here to stay and that dental schools must engage with it now to realise its potential; and recognition that educators do not know enough about how students are using AI and need to collaborate with our students in future development and research.

## Key points

- This paper highlights the most pressing challenges and opportunities of AI in dental education.
- Provides a focus for future collaborative research.
- Encourages educators to engage with AI for its positive aspects.

## Background

Artificial intelligence (AI) has been defined as ‘the science and engineering of making intelligent machines'.^1^ Since the term AI was first coined at the Dartmouth workshop in 1956, it has become increasingly embedded in many aspects of modern life.^2^ In 2022, Stephen Marche made concerning predictions about the impact of AI in general academia and described an urgent need for UK dental education to get ‘with it' and develop an AI strategy; his declaration that ‘The college essay is dead' and belief that the use of AI would ultimately undermine the pedagogical design of education sent a ripple of anxiety throughout academia.^3^

Recent debate seems to have focused largely on the challenges associated with the use of AI, rather than the opportunities it may afford. In dentistry, AI is already being used to support both clinical and education practice, for example, diagnostic and planning purposes within clinical practice, while in education, AI is being used to automate repetitive administrative tasks, develop content and provide intelligent tutoring systems. Many educators, however, have limited knowledge or skills in the use of AI, while technology has and continues to evolve exponentially.^4^ In dental education, it is suggested that it will take two years for students to become proficient in AI use, three years for academics to realise that students are using this technology, and a further five years for ‘faculty' to decide what to do about it.^4^

## AI conference

The British Alliance of Researchers in Dental Education and Scholarship (BARDES) hosted an AI-themed conference in Glasgow in November 2023. One of the primary objectives of BARDES is to facilitate connectivity of dental education providers to identify, enable and enhance delivery of educational research. Recognising the urgent need to initiate research and evidence-based development within the sphere of AI, the conference organisers set out to provide insight into different perspectives of AI from practitioners in the field of AI, ethics and dental education, and initiate general discussion on the use of AI in UK dental education. The conference included a structured workshop to initiate wider enquiry of the subject.

In order to deliver a more focused and meaningful discussion before the conference, the four workshop leads (who were all experienced dental educators based at Newcastle University) met with local AI experts to ‘brainstorm' a list of potential opportunities and threats that exist with the adoption of AI technologies in education. The intention was to provide prompts for onward discussion. The brainstorm was conducted in an informal way by virtue of an open discussion of perceived opportunities and threats. This identified nine potential opportunities and six potential threats (see Table 1).

**Table 1:** Full list of potential opportunities and threats that could exist in relation to the use of AI in dental education in the UK

**Potential opportunities**	Drive efficiency and increased productivity of educators (the use of tools to support lesson planning and content creation Drive innovation and development of high-quality teaching and assessment (delivery of personalised formative assessment and feedback, and simulation and skills development such as creation of human-like virtual patients) Equality (supporting those with a disability and whose primary language is not English) Facilitate personalised learning, student support and self-study (support, guidance and information bots, personalised learning plans and journeys) Development of critical thinking and evaluation skills (appraisal of AI-generated text) To promote appropriate use of AI by students (appreciation of how AI should be used to facilitate their learning and how it should not be misused) Impetus for change Enhanced engagement by students Benefit to patients (AI to enhance communication with ability to convey the same information in the style, level and language required for that patient, patient management) Other.
**Potential threats**	Misuse (potential for academic misconduct, malicious impersonation of others) Data security/privacy (IP of any input and output into an AI tool) Inequity in training/capability (awareness, willingness and ability to use AI tools effectively) Role of the educator (potential for AI tools to change what we do and how we do it, possibility that it might make teachers redundant) Inequity of access (those that can pay and/or can keep up with developments could access better AI tools) Don't know enough about it (‘fear of our technological limitations' averse to change and adopting new approaches and new technologies) Other.

An anonymous questionnaire listing the nine potential opportunities and six potential threats was then created using Microsoft Forms (Microsoft Office 365 Education) and sent via email to the 71 conference attendees who had registered at that point, one week in advance of the conference. Participants were asked to rank the priority of these opportunities and threats and were also given the opportunity to suggest any other additional opportunities or threats.

There was a 38% response rate (27 responses were received). Data were analysed using the Microsoft Forms (Microsoft Office 365 Education) automated function, which calculated the average ranking for each statement from the ‘weight' of the ranked position (where an option ranked first will have highest weighting and the one ranked last will have the lowest) and the number of times a statement was chosen. This analysis technique provides the most preferred statements overall. The ‘top four opportunities and top four threats' were then taken forward into the workshop during the BARDES conference. These were:

- Opportunities:
  - Drive innovation and development of high-quality teaching and assessment
  - Facilitate personalised learning, student support and self-study
  - To promote appropriate use of AI by students
  - Equality.
- Threats:
  - Misuse
  - Data security/privacy
  - Inequity in training/capability
  - Inequity of access.

The opportunities and threats were used as prompts to initiate conversations around eight separate facilitated tables. Each table was facilitated by an experienced dental education researcher who was also experienced in the leadership and management of dental programmes. The facilitators represented Newcastle, Glasgow, Liverpool and King's College London. To stimulate discussion, facilitators were provided with three open questions to use as prompts:

- What do you mean by AI?
- What is your school's current practice/experience of this aspect of AI?
- In relation to this aspect of AI, what question needs further exploration in order to realise opportunities and/or manage any threats?

The discussions were recorded manually by the facilitators and then transcribed and annotated by the same facilitator. The outputs from each facilitator were collated by the workshop leads. The leads reviewed and reflexively discussed the transcripts to identify key areas.

The 76 workshop attendees were affiliated to 14 dental schools across the UK and Ireland, as well as NHS England, Dental School's Council, General Dental Council, primary dental services and hospital dental services.

Ethical approval for this work was gained from Newcastle University (ref 45804/2023). Before completing the pre-conference questionnaire and attendance at the workshop, participants were informed of the purpose of the study and provided with the opportunity to withdraw from completion of the questionnaire and or the workshop.

## Opportunities

### Personalised learning

In terms of opportunity, workshop participants identified that the ability of AI to support personalised learning for students was a priority area to explore.^5^ This emerged through several of the groups but had not been a previously suggested as an opportunity in its own right. Personalised learning or development plans are inextricably linked to student monitoring in terms of academic achievement and clinical experience. The meaningful analysis of big datasets of student performance (eg from clinical logbook and formative assessment in e-portfolios) to predict future clinical performance and progression, subsequently enabling tailored clinical training/teaching, such as adaptive clinical management/patient allocation and timetabling, were identified as areas where AI could be particularly helpful. Accurate, meaningful analysis which could occur in real time would allow for timely identification of students who were not fulfilling their potential, to enable the academic team opportunity to put in place appropriate support and/or remediation. Research has also suggested that reactive and predicative feedback benefits student motivation and outcomes in non-clinical education.^5^ The time academics have previously spent in gathering and understanding a large dataset could be transferred to providing support or developing other educative resources for the students, meaning that more time is available to directly benefit the student. Enhancing data gathering and analysis would also help to develop a robust evidence base to test existing dogma around development of skills.

A further opportunity discussed for personalised learning was the ability of AI to generate student-tailored resources that address their preferred method of learning, for example generation of tests, practice papers, objective structured clinical examination stations, mind maps, audio recordings etc.

### Supporting learning

Along similar lines, but with specific reference to students with learning disabilities and/or additional learning needs, AI was seen as having the potential to record, transcribe, or synthesise bullet-pointed learning material, or could be used to ‘jump start' a conversation or email with a member of staff. This would be a useful tool in assisting students with a disability or disadvantage, helping to level the educational playing field. Intellectual tutor systems (ITSs) have been described as the most common use of AI in education.^6^ This broad term includes the delivery of sequential information through clicks or activities (such as quizzes). Research methods tutoring (RMT) has been described as an ITS with one-to-one dialogue to help the student develop higher level knowledge and understanding and has been shown to be effective in psychology.^7^ Such tools can be developed to specifically support those with learning disabilities and this adaptation of AI has shown promise already.^8^

Student wellbeing is an area of academic provision that is becoming extremely resource heavy and delegates talked of ‘safe' support ‘apps' that could be helpful to manage overwhelming student demands on wellbeing services as an example There are currently university-endorsed services such as SilverCloud (Amwell), an online cognitive behavioural therapy tool which can be tailored to a student's specific needs.^9^^,^^10^ Other areas that could be delivered by AI included supporting students with managing workloads by weekly diary planning.

Interestingly, participants also questioned whether interactivity with AI which enabled ‘emotion-free' feedback could be something students may perceive as beneficial, potentially providing a greater level of ‘psychological safety' in a world where students seem afraid to answer questions in ‘public' for fear of getting the answer wrong. However, this also raises further questions and could present other challenges beyond the scope of this paper.

### Educator resources

Perhaps one of the most anticipated opportunities explored was the ability of AI to reduce staff workload in relation to the preparation, marking and analysis of educational material, thus freeing staff time for more hands-on teaching. It was thought that AI generation of questions and simulated patient interactions could provide a wider range of diversity at a lower cost compared to role players, for example, while also being able to screen out biases. The counter arguments to this use of AI was concern that generated material might include inaccurate images or materials, and that educational or assessment materials produced may have a western bias due to the prevailing origin of AI source material. It was also postulated that students might query the value of their education if they thought content was significantly AI-generated.

It was felt that there was a significant opportunity and need to educate students over the appropriate use of AI, for example, understanding the limitations of integrating AI with evidence-based work and the need for critical thinking and review. For many delegates, this hinged around having robust regulations, ethical use guidelines, linkage to professional identity and transparency of use.

### Equality

One of the key areas of discussion was around equality, where AI was thought to have some significant advantages but also problematic disadvantages. AI was seen to enhance the availability of information to all and provide ‘expert' input in situations where students may not have access otherwise - an example being a candidate applying to study dentistry who has no family/friend connection to dentistry or limited access to careers advice.

The use of AI in dental diagnostics and treatment planning was also discussed. Given that curriculum content would need to change to include these, it was thought that a cohesive approach from all stakeholders would be beneficial. AI-generated images of unrealistic patient outcomes may also be problematic in raising unrealistic expectations for both patients and clinicians.

## Threats

### Digital literacy

Access to devices was not seen as an obvious source of inequity, given that all students are likely to have access to a smart phone, laptop or on-campus desktop computer. However, inequity was suggested to exist around access to, or the ability to purchase, the most up-to-date licenced products. The COVID-19 pandemic and emergency move to online learning highlighted the inequity of digital literacy.^11^ Institutional licence purchase could be valuable in this regard and students could be instructed to access the same, reliable/approved AI site. There was, however, a general consensus that student access to a high specification personal desktop or laptop with appropriate WiFi connectivity may not be universal.

The greatest source of inequity discussed was in relation to training and capability, where perceived variation between students and staff, among staff, and among students might exist. Experience with AI before attending university was highlighted as an area subject to inequality due to variability among schools.^12^

### Misuse

Perhaps the area that raised most concern was potential misuse of AI; it was postulated that a stakeholder could potentially use AI systems to gain an unfair, misleading, or unethical advantage by intentionally misrepresenting their abilities. Misuse could include academic misconduct, profiteering, patient harm, data security, and the provision of inappropriate educational processes.

Academic misconduct was looked at from the perspective of both staff and students. For staff, AI could be used to generate papers, learning support material, and assessments to claim as personal intellectual property to facilitate promotion. Regarding students, academic misconduct might include cheating, plagiarism or ‘malicious interpretation'. Examples of malicious interpretation could be allowing an imposter to take an assessment, or the creation of a false identity or persona to engage with an admissions process.

Wider world/industry profiteering may be beyond the scope of higher education institutions to manage, but interesting questions were asked about who is profiting from AI and what the motivation is of big industries. Is there unknown/unclear manipulation going on and who is behind it (false news, political gain, industry dominance)? Are there vulnerabilities that AI may exploit to undermine experts or the knowledge base and therefore affect evidence-based healthcare?

However, even on a smaller individual scale, those with highly developed AI skills may gain advantage over others or attempt to sell their skills onto others. Interestingly, several of the delegates expressed their fear about not knowing enough, and becoming obsolete, while others expressed feelings of shame for using AI to generate material as they felt it was a form of cheating.

### Safety

In the longer-term, misuse of AI could result indirectly in patient harm through its potential impact on learning and learning skills. In the first example, concerns were expressed about the use of AI preventing/inhibiting students from engaging with knowledge, understanding and failing to build interest. Secondly, if used in such a way that was blind to the limitations of AI, there was potential for learners to fail to develop helpful thought and synthesis skills. There was concern that, over time, there could be a reliance on AI and loss of natural clinical and communication skills. There was also concern about a more direct cause of patient harm when, for example, asking AI for a clinical solution which is then wrong and results in patient harm.

Threats around data security and safety were thought to exist and discussions touched on the use of AI in a clinical setting (consent, previous records, ownership of data, access to records, how the inputs or feedback from systems are being used), the problem of treatment plans being produced by AI and justification of clinical decisions made on the information generated by AI. There was a consistent viewpoint that human input is still needed and AI is an adjunct, not a replacement.

The discussion indicated that, currently, there is a diverse approach in UK dental schools regarding AI, with a focus largely on assessment risk management. This focus was almost exclusively linked to academic anomalies or misconduct, justifiable given that assessments represent thresholds to student progression and ultimately graduation and professional registration. By and large, dental schools tended to fall in with wider institutional policy without any cohesive strategy or sharing of practice and experience.

## Strengths and limitations

The purpose of the workshop was to explore areas of AI usage that are recognised by UK dental education providers as being of priority for collaborative research. This was in line with one of the primary objectives of BARDES, which is to facilitate connectivity of dental education providers to deliver educational research.

The pre-conference questionnaire aimed to create a manageable number of possible themes as prompts to discussion. While a response rate of 38% could be considered to be quite low, it is in keeping with expectations of this type of research tool in this group of individuals.^13^ Moreover, the ranking developed by the questionnaire was only ever intended to provide prompts and did not exclude wider discussion; indeed, a number of ‘new' themes emerged during the workshop, indicating that this approach had been successful.

As an example, patient safety had not been identified as a ‘threat' in either the initial brainstorming or by using the ‘other' option during completion of the pre-conference questionnaire and yet, the nature of workshop table discussion enabled this to be uncovered and explored.

As a qualitative strategy was employed, there was never any intention to analyse the percentage of schools and/or participants that identified the above themes as being an opportunity or threat; however, this exploration now leads the way for further research that could look at this issue from a more quantitative perspective, from both the view point of education providers and their consumers.

## Conclusion

There were two key areas that came up time and time again. Firstly, the recognition that AI is here to stay and that dental schools must engage with it now to realise the diverse opportunities it presents. At the moment, the focus seems to return more to threats and uncomfortable uncertainty. It will therefore be essential to ensure a balance with the opportunities that are exciting and, in the eyes of the authors, worth pursuing. Secondly, the recognition that we simply do not know enough about how students are using AI and how they view it; therefore, in order to undertake meaningful research and development work around AI, we must speak with our students and aim to co-create future AI-focused curricula and resources.

## Data Availability

The data that support the findings of this study are available on request from the corresponding author. The data are not publicly available due to their containing information that could compromise the privacy of workshop participants.
